# Sensors and Instruments for Brix Measurement: A Review

**DOI:** 10.3390/s22062290

**Published:** 2022-03-16

**Authors:** Swapna A. Jaywant, Harshpreet Singh, Khalid Mahmood Arif

**Affiliations:** 1 Department of Mechanical and Electrical Engineering, SF&AT, Massey University, Auckland 0632, New Zealand; s.jaywant@massey.ac.nz; 2 New Zealand Product Accelerator, The University of Auckland, Auckland 1010, New Zealand; h.singh@auckland.ac.nz

**Keywords:** Brix sensor, sugar content, agricultural products, fruit quality, alcoholic beverages

## Abstract

Quality assessment of fruits, vegetables, or beverages involves classifying the products according to the quality traits such as, appearance, texture, flavor, sugar content. The measurement of sugar content, or Brix, as it is commonly known, is an essential part of the quality analysis of the agricultural products and alcoholic beverages. The Brix monitoring of fruit and vegetables by destructive methods includes sensory assessment involving sensory panels, instruments such as refractometer, hydrometer, and liquid chromatography. However, these techniques are manual, time-consuming, and most importantly, the fruits or vegetables are damaged during testing. On the other hand, the traditional sample-based methods involve manual sample collection of the liquid from the tank in fruit/vegetable juice making and in wineries or breweries. Labour ineffectiveness can be a significant drawback of such methods. This review presents recent developments in different destructive and nondestructive Brix measurement techniques focused on fruits, vegetables, and beverages. It is concluded that while there exist a variety of methods and instruments for Brix measurement, traits such as promptness and low cost of analysis, minimal sample preparation, and environmental friendliness are still among the prime requirements of the industry.

## 1. Introduction

**Figure 1:**
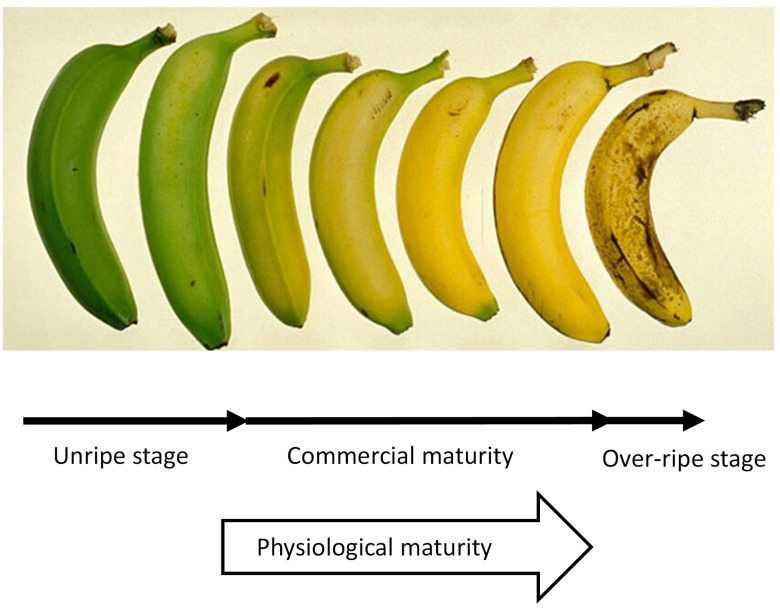
Different stages of fruit maturity.

Fruits and vegetables play a vital role in the human diet as they are rich in carbohydrates, minerals, vitamins, dietary fibres, and antioxidants. The crop quality can be evaluated by various attributes such as appearance, texture, and flavor. The appearance factors are size, shape, and color whereas the factors associated with texture include firmness, crispiness, and juiciness. The flavor is defined by sweetness and aroma. However, harvest maturity is the crucial factor determining the shelf life and final quality of any fruit or vegetable [1,2]. The different stages of harvest maturity are shown in Figure 1. Sugar content is one of the measures used to judge the various stages of maturity [3]. Hence, it is the pivotal factor in agriculture, the food industry, winemaking and brewing. It is a significant indicator in harvest scheduling, postharvest management, and variety selection for farmers. In the food industry, to produce different soft drinks, tomato concentrates, fruit juices, and honey, sweetness control is the prime element. In a winemaking and brewing, the fermentation process converts sugar into ethanol (alcohol) and carbon dioxide with the help of a microfungus called yeast. The measurement of sugar levels in a grape must or a wort allows winemakers and brewers to estimate the alcohol content after the fermentation. This calculation is an essential part of winemaking and brewing that can affect decisions made in the alcohol industry. Dissolved sugar in the fruit juice is measured in terms of Brix, Baume or Oechsle. However, the most widely used unit in the food and beverage industry is Brix. Brix can be measured in the field, on a plant, or at a shop. It indicates the number of dissolved solids in a liquid measured via its specific gravity (SG). One degree Brix is 1 g of sucrose in 100 g of solution (1 ${}^{\circ }$Brix = 1% sugar) [4,5,6,7,8].

Since sugar concentration has a linear relationship with SG and density, Brix can be measured via any of these two quantities. The most popular instrument for Brix measurement is either refractometer or a hydrometer, where a refractometer is an optical device, and a hydrometer is a glass device used to measure the specific gravity [4,5,8]. There are various other methods to measure Brix value such as differential measurement, density measurement, osmotic potential measurement, mass flow measurement, optical, ultrasonic, and biosensors. Brix measurement methods used in agriculture and horticulture commodities are generally categorized as destructive and nondestructive types. In the destructive-type method, fruit extract is required to perform the measurement, whereas, in the nondestructive methods, Brix measurement is performed on the fruit without cutting it. Brix measurement methods used for the process industry can be classified as in-line measurement and sample-based measurement methods. The sugar content analysis of fruit and vegetables using destructive methods include sensory assessment involving sensory panels, using instruments such as a refractometer, hydrometer, and liquid chromatograph [9]. However, these techniques are manual, time-consuming, and, most importantly, the crops (fruits and vegetables) are damaged. In the winery, the traditional sample-based methods require sample collection of the liquid from the tank. In a commercial winery, this is a mundane job that involves several man-hours. Therefore, an automatic and continuous Brix measurement can always be an asset to the wine industry. Hence there is a continuous demand for a real-time, rapid, noninvasive, and continuous monitoring approach for the sugar content analysis [9,10].

In this paper, we present different destructive and nondestructive methods for the determination of Brix in the farming industry, along with the advantages and disadvantages of each. Destructive detection covers methods using tools such as a refractometer, hydrometer, electronic tongue, lossy mode resonances, and SPR-based sensors. Nondestructive detection covers visual and near-infrared spectroscopy and magnetic resonance methods. In addition to this, sample-based Brix measurement methods and commercially available in-line measurement techniques used in wineries and breweries are also discussed, highlighting the advantages/disadvantages and limitations.

## 2. Sample-Based Destructive Methods for Brix Measurement

All the methods discussed below require a juice sample of fruit or vegetable to perform Brix measurement. Hence these are classified as destructive methods in the farming industry. Due to the sampling nature of these techniques, they are also considered to be sample-based techniques, particularly in the process industry.

### 2.1. Hydrometer

**Figure 2:**
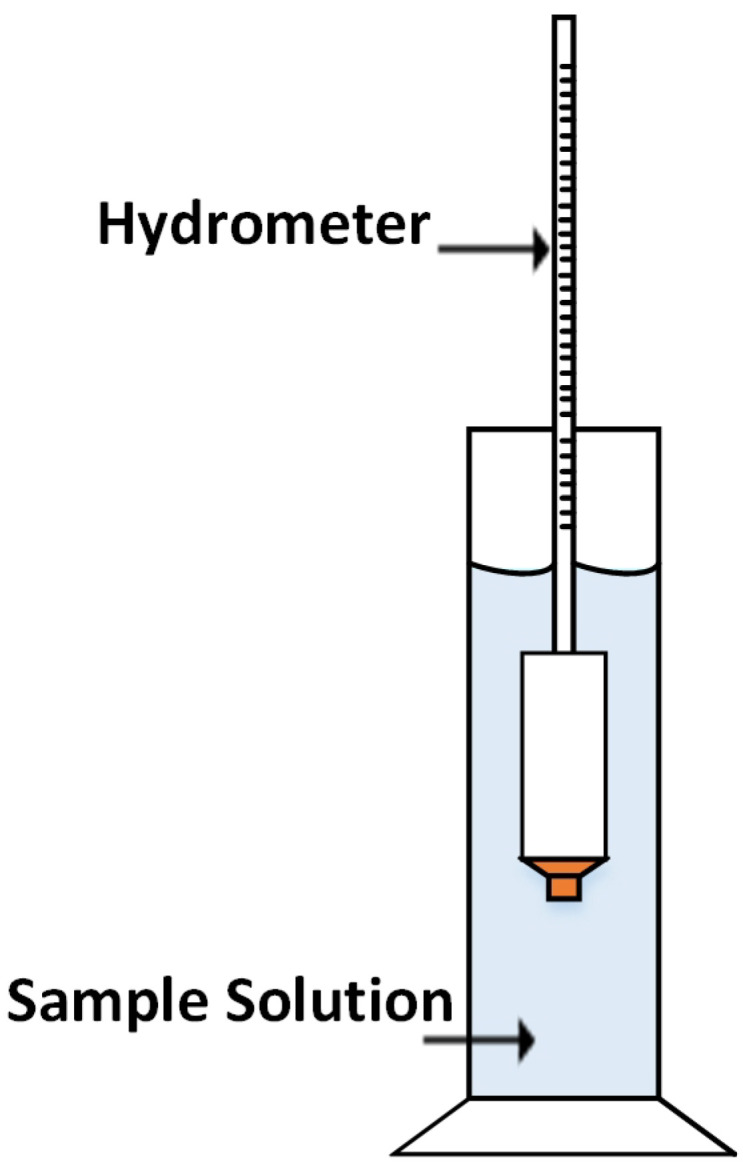
Brix measurement with hydrometer in a graduated cylindrical container.

A hydrometer is a traditional instrument that measures Brix in terms of SG. It works on the concept of buoyancy and is usually calibrated at room temperature (20 ${}^{\circ }$C) in different units such as ${}^{\circ }$Brix, Baume, and SG, as per the requirement [9,11,12]. The working of hydrometer is based on Archimedes’ principle, which states that “the upward buoyant force that is exerted on a body immersed in a fluid, whether fully or partially, is equal to the weight of the fluid that the body displaces”. Hydrometer consists of a wide-bottom (for buoyancy) sealed hollow glass tube, a lead or mercury ballast for stability, and a graduated narrow stem for measurement as shown in Figure 2. Typically, a hydrometer is lowered into the liquid filled graduated cylinder until it floats freely. The point where the surface of the liquid touches the hydrometer’s stem is proportional to the relative density of the liquid.

The use of a hydrometer is one of the most widely used techniques to read the Brix value in the food and farming industry, and wineries and breweries. However, this method is unreliable as it involves a manual operation that can lead to errors such as the incorrect reading of the meniscus. Moreover, temperature compensation is required as the operation is dependent on temperature variation. Sometimes, surface contamination occurs due to the presence of insoluble particles in the liquid and causes a substantial difference in the surface tension, resulting in an additional error [9]. To overcome these inaccuracies, another optical tool known as refractometer is more widely used to measure the sugar level.

### 2.2. Refractometer

A refractometer is an optical instrument to measure the Brix level in fruits, vegetables, juices, jams, wines and beers. It is a standard technique based on the measurement of the refractive index of the liquid. Refractive index (RI) is a ratio of the speed of light in a vacuum to the speed of light in the given medium. RI depends on the wavelength of the light and the temperature of the sample liquid [13,14]. Different types of refractometers commonly used in fruits and vegetables research are listed in Table 1 to provide a source of quick information for the readers. Most of the refractometers are either based on the principle of refraction or the principle of critical reflection of light. According to [15], critical angle-based RI measurement is more appropriate as it is not influenced by the colour of the sample liquid or the suspended solids in the sample liquid. As a result, a refractometer can be conveniently used to perform quality checks in industries such as food and dairy [13].

Refractometers are available as digital or analog types, in handheld and bench-top versions. Similar to a hydrometer, an analog refractometer needs temperature correction while performing the measurement. However, Ugwu et al. [14] have fabricated a handheld analog refractometer that includes an in-built temperature compensation. This refractometer consists of focus adjustment, calibration screw, daylight plate, eyepiece, rubber grip, and the main prism assembly. A charge-coupled device (CCD) has been used as the sensor to precisely measure the intensity of the reflected light. The refractometer must be calibrated before taking measurements with the help of a calibration screw and distilled water for temperature compensation. The temperature-compensation thermometer is built within the refractometer. The researchers conducted the test using samples of orange, pineapple, and cashew juices at certain levels of pH values. A significant drawback of such a handheld analog refractometer is that a person takes reading at a time that may be subjective.

Digital refractometers perform temperature compensation automatically. However, such refractometers usually display errors if readings are taken too quickly before a temperature equilibrium is reached [9]. The benchtop devices can provide an accurate reading with automatic temperature control from 0 to 100${}^{\circ }$ along with quality control analysis. They are readily available, robust and not influenced by ambient conditions. Compared to benchtop devices, handheld devices are cost-effective and portable. The most common commercially available refractometers are summarised in Table 2, along with their specifications.

**Figure 3:**
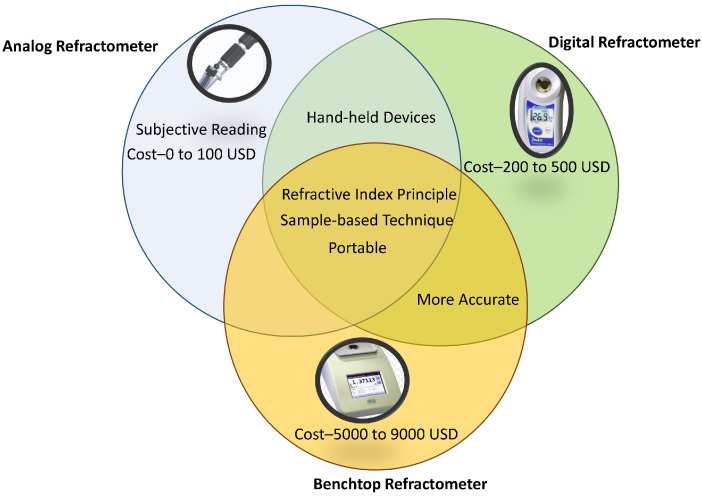
Features of analog, digital, and benchtop refractometers.

Figure 3 pistorially describes the advantages and limitations of different types of refractometers (analog, digital, and benchtop refractometers). Advantages of analog refractometers include portability and cost-effectiveness, whereas the subjective nature of their readings is their major limitation. Digital refractometers are handheld, cost-effective, and more accurate compared to analog refractometers. Benchtop refractometers are more accurate compared to analog and digital refractometers, however, they are more expensive than other types of hydrometers.

### 2.3. Other Refractive Index (RI)-Based Destructive Methods

Dongare et al. [13] have developed an RI-based optical Brix meter using an equilateral angle prism. The optical configuration of the developed system includes the use of LED (light-emitting diode) as a source and LDR (light-dependent resistor) as an optical detector. The variation in concentration of sugar causes variation in the RI, and, therefore, the critical angle of the total reflection changes. This, in turn, produces a change in the LDR resistance. The range of the Brix meter is from 1 to 35 ${}^{\circ }$Brix. The possibility of another RI method to measure the sugar content has been introduced by Seki et al. [15]. They have investigated RI measurement in sucrose solution with the help of a heterocore-structured fibre optic-based SPR sensor. The sensor was developed by depositing a silver thin film on the surface of a heterocore-structured fiber optic. The result obtained by the sensor confirmed an excellent alignment with the conventional refractometer readings. Furthermore, Zubiate et al. [32] fabricated a Brix sensor that can be used in the real-life applications. The working principle of the sensor is based on Lossy-Mode Resonances (LMRs). They deposited thin indium tin oxide (ITO) films onto D-shaped optical fibers. The sensor can monitor from 0 to 50 ${}^{\circ }$Brix. The device has a linear response with a linearity factor R^2^ 0.9961 and 11.8 nm/${}^{\circ }$Brix sensitivity (~0.005 ${}^{\circ }$Brix resolution).

### 2.4. Electronic Tongue

One of the conventional methods for quality measurement of fruit, vegetables, different types of juices, and wines is the use of a sensory panel [33]. In this method, a qualified group of panelists evaluates the product’s taste based on different qualities such as sweetness, sourness, bitterness, and aroma. The study is always influenced by subjective readings, the lack of standard methods to rank the product, problems of stability, and reproducibility. It is a time-consuming method and also involves a high cost [9,33,34]. Nevertheless, this has inspired many researchers to develop an electronic device (commonly known as an electronic tongue) that can be utilized as artificial human taste buds.

**Figure 4:**
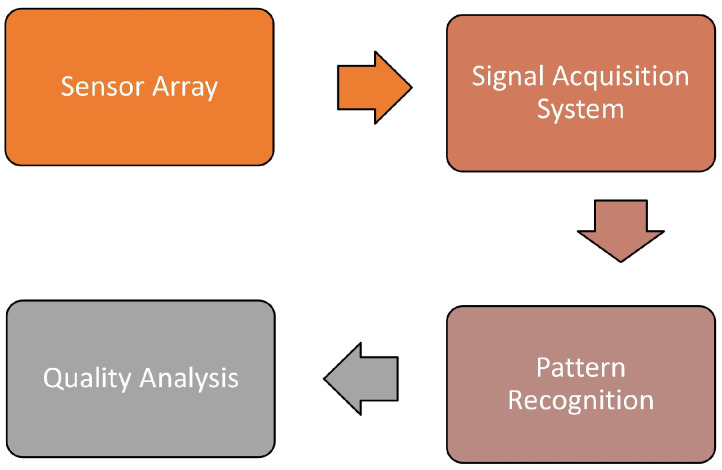
Constructional block diagram of an electronic tongue.

An electronic tongue utilizes an array of sensors based on potentiometric, voltammetric, impedance spectroscopy, and piezoelectric working principle in conjunction with different pattern-recognition methods as shown in Figure 4 [35,36,37,38]. However, the potentiometric principle is more commonly used than the other principles. It makes use of ion-selective electrodes to measure the potential change against a reference electrode in zero-current conditions. Low cost, rapid response, and easy-to-handle measurement setup are the main advantages of any electronic tongue.

Toko and coworkers were the pioneers of the electronic tongue [35]. Since then, many research groups have widely used many electronic tongues for quality analysis of fruits, vegetables, fruit juices, soft drinks, tea, coffee, alcohol, and herbal products. Beullens et al. [39] used an electronic tongue to determine the sugar and acid profile of four tomato cultivars. An array of 15 potentiometric sensors were checked against the high-performance liquid chromatography (HPLC) result. To study the potential of the electronic tongue to predict the concentrations, partial least squares (PLS) regression models were built. PLS study was undertaken using the results of the electronic tongue data and the HPLC measurements. However, the sensor was found to be less sensitive during sugar measurement as it only achieved a correlation of 0.69, 0.53, and 0.63 with sucrose, glucose, and fructose, respectively. Bett-Garber et al. [40] demonstrated the possibility of using an electronic tongue to monitor processed juice quality by comparing six different brands of pomegranate juice. In another study [41], an electronic tongue was applied for monitoring the consequences of postharvest techniques on the fruit-ripening process. In the comparison of instrumental analysis and sensory analysis it was found that the instrumental results were more sensitive to differences than the sensory analysis. Furthermore, Campos et al. [42] demonstrated the capability of an electronic tongue to measure the grape ripeness parameters (Total Acidity (TA) and ${}^{\circ }$Brix) parameters that are useful to estimate the harvesting date. They used voltammetry along with metallic electrodes. Similarly, Lozano et al. [43] fabricated a similar electronic sensing device to identify different wines’ ageing processes. Pattern recognition has been performed using both linear techniques such as principal component analysis, (PCA) and nonlinear techniques such as probabilistic neural networks (PNN). These systems achieved a classification success rate of 97% with PCA and 84% with PNN during the detection of different ageing processes. All these researched RI-based sensors are summarized in Table 3.

## 3. Nondestructive Methods for Brix Measurement in Fruits and Vegetables

### 3.1. vis/NIR Spectroscopy

In the farming industry, visible and near-infrared (vis/NIR) spectroscopy demonstrate significant potential for sorting and grading fresh produce as per their internal attributes [10,44,45]. Over the last 2 decades, across the world, a large number of studies have been conducted to predict the sugar content in various fruits and vegetables with the help of vis/NIR techniques [44,46,47,48,49,50,51,52,53,54,55,56,57,58]. The method uses visible and NIR range of the electromagnetic spectrum, covering approximately 300–2500 nm to measure the sugar content of different fruits such as apples, bananas, pomegranates, kiwifruit, mandarins, mangoes, oranges, pineapple, sugarcane, and tomatoes (Table 4). The NIR spectral acquisition from the sample is performed with the help of three standard modes—transmission, reflectance, and interactance [59]. In the transmission mode, while infrared light is passing through a sample, some part of the light can be absorbed by the sample and the remaining fraction is measured on the other side of the sample. Alternatively, light can be reflected from the sample and the absorption properties can be obtained from the reflected light in the reflectance mode. In the transmission mode, a light source and a light detector are placed on either side of the sample, whereas in reflectance mode the source and the detector are placed on the same side of the sample. The interactance mode is similar to the reflectance mode, however, the detector is separated from the illuminated sample by the light seal [60]. In the case of reflectance mode, the spectral acquisition is easier with a relatively high-intensity level, but the shortcoming of this mode is that it is prone to the variations in shallow subsurface or on-surface properties of the fruit; on the other hand, the transmission mode may be less sensitive to surface properties, but it has a reduced signal-to-noise ratio as the amount of light penetrating the flesh is very small [60]. Hence it becomes difficult to pick up accurate transmission readings at grading line speeds, especially where high surrounding light is present. Interactance mode can sort out the problems related to reflection and transmission modes. Hence it can be the most effective type of analysis. However, this mode requires proper light sealing, which is challenging with the high-speed conveyors used in current fruit-sorting systems [44].

To evaluate the Brix value of a variety of apple fruits Angra et al. [61] used a filter-based NIR technique where the in-house developed probe recorded the reflectance wavelengths. A standard error of prediction for Brix values of all varieties of apples was between 0.7322 to 1.7809. Sripaurya et al. [46] developed a portable 6-digital-channel NIR device to predict the quality of Gros Michel bananas and grade them by maturity stage. They also proposed a novel automatic classification of banana-ripening classification results, they where classified up to seven ripeness levels with nearly 100% accuracy. Liu et al. [49] explored the viability of the CCD-NIRS (charge-coupled device—near infrared spectroscopy) technique combined with interval partial least squares (iPLS) algorithm for measuring the soluble solid content (SSC) and TA of Nanfeng mandarin fruits. The author observed the improved predictive ability of the SSC model with r of 0.92 and RMSEP (root mean square errors of prediction) of 0.65 ${}^{\circ }$Brix using effective wavelengths of 681.36–740.51 nm, 798.60–836.19 nm, and 945.52–962.75 nm. Golic et al. [62] projected glucose, fructose, and sucrose in citrus fruit through reflectance vis/NIRS technique. The predictability of glucose and fructose was much more than sucrose in this analysis. The authors thought that the difference in the molecular weights of sucrose (MW = 342.30 g/mol) when compared to glucose and fructose (MW = 180.16 g/mol) may be a possible reason for the lower predictability of sucrose. The number of sucrose molecules is 1.89 times fewer than glucose and fructose molecules in the same weight of the sample.

Though a nondestructive NIR spectroscopic approach looks promising for the quality analysis of fruits, it requires complex and expensive instrumentation [50]. A few groups attempted successfully to create an in-house portable NIR system to counter the cost of the commercially available systems [50,51,52,53,54,55,56]. Saranwong et al. [55] compared the performance of a commercially-available, portable NIR instrument “FT20” with a research-type spectrophotometer for fruit-quality evaluation using mango fruits and found similar accuracy in both.

Even if the spectroscopic technique is considered the most viable method for nondestructive quality analysis of fruits and vegetables, it still has some limitations. The major disadvantage is that the method is based on reference values that may be susceptible to errors. It requires a new calibration model for every fruit species. Regarding changes in season and location, it is desirable to upgrade calibration models according to these [63]. Still, with the recent developments in vis/NIRS techniques, the technology has great potential in the industry for sorting and grading the produce.

### 3.2. Magnetic Resonance Methods

Magnetic resonance imaging (MRI) is a noninvasive method based on nuclear magnetic resonance (NMR) principles. It can provide highly resolved spatial information related to the distribution and environment of water in soft tissues [64]. The molecular elements of fruits consist of magnetic resonance (MR) active nuclei that can be utilized to sense the different quality attributes of the fruits, such as texture, taste, internal structure, Brix number [65]. MR attributes such as proton density, relaxation times, chemical shifts, and diffusion constants have the potential for quality analysis of fruits. The relaxation times can be utilized to assess the fruit-ripening stage, fruit firmness, as those are sensitive to mobility. A time-domain analysis is used in the study of relaxation and diffusion data [66]. Table 5 summarises the use of relaxation time for different applications reported in various research.

Andaur et al. [71] studied the growth and ripening of grape berries for three varieties using MRI. Their study confirms that it is possible to visualize the internal features of berries with such a nondestructive technique and to acquire the information related to volume and ${}^{\circ }$Brix distribution within a cluster. Zhang and McCarthy [67,68] used the MRI technique to investigate the quantitative analysis of pomegranate quality and tomato maturity. They could relate the soluble solids content of pomegranate with Spin–spin relaxation time, T2, measured using a low magnetic field (0.04 T). Similarly, a detailed study of biophysical, histological, and metabolic traits of fruit development was reported by Musse et al. [69] with a dependable explanation of the changes in relaxation parameters. Hence, MRI can be used to provide information on cell expansion in a noninvasive manner.

A few portable, dedicated MRI equipment are available for various applications in the food and farming industry. Additional attempts at miniaturization are in progress by using microfluidic technology [72] and integrating all the required RF electronics into a single board. Such efforts have been demonstrated by Sun et al. [73]. Windt and Blumler [74] have developed a portable NMR sensor to measure the dynamic changes in fruits.

Even though NMR spectroscopy and MRI have been proved to be an effective nondestructive method for the quality evaluation of fruits, they have found only limited applications in modern industrial and commercial applications. The size, weight, cost, and stability of magnets and the throughput are the major constraints for the wider application of MR-based technology [66]. The specifications of nondestructive Brix sensors are summarized in Table 6.

## 4. Brix Measurement in Winemaking and Brewing

**Figure 5:**
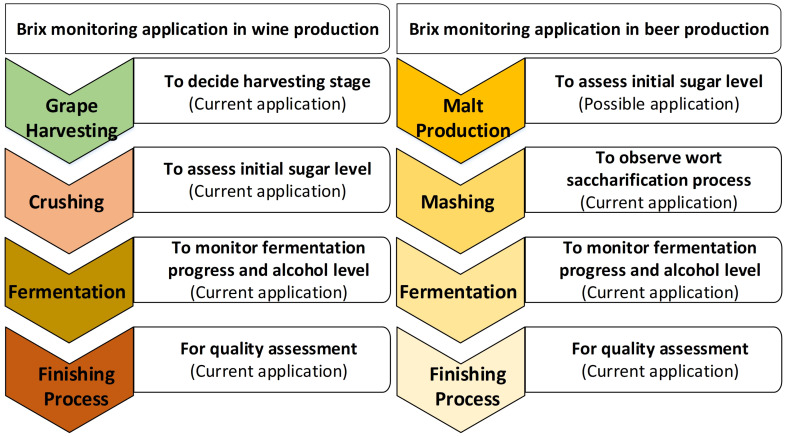
Brix monitoring at different stages of wine and beer production.

In wineries and breweries, optimised process control is the prime factor for enhancing wine or beer quality. This includes the quality assessment of physical properties of raw materials (e.g., grape juice/malt), process streams (e.g., ferments), and end products (e.g., wine/beer) [75]. The fermentation process control is crucial during the production of wine or beer to monitor the conversion from raw juice to alcohol [76]. It is important to observe the Brix level at the various stages of beer and wine production (Figure 5). In this section, different types of Brix monitoring methods that are used during the production of wine and beer are discussed.

### 4.1. Spectroscopic Methods

Over the last few years, it has been found that vis-NIR spectroscopy can be a potential method to observe the conversion process and anticipate the concentration during wine fermentation in real time [77,78]. Currently, in the winery, the principal application of this method is the calculation of alcohol and Brix content with the help of modern filter instruments such as monochromator and diode array spectrophotometers together with advanced algorithms and software for wine analysis [78]. It has been reported that many big Australian wine companies are utilizing NIR spectroscopy to perform composition analysis of red wine [78]. Investigation trials during the wine fermentation process also revealed that these methods could predict the concentration and monitor the extraction and evolution of phenolic compounds during red wine fermentation. Analysis of different wines using vis-NIR spectroscopy by various authors is summarised in Table 7. Similarly, Grassi et al. [79] demonstrated the possibility of FT-NIR spectroscopy combined with multivariate data analysis to monitor and assess process parameters in beer fermentation at different operative conditions.

### 4.2. Ultrasonic Methods

For the past few years, ultrasonic-based instruments have been used to control a variety of industrial processes. These days, ultrasonic technology is considered to be a relevant method to monitor quality control in food processes due to its qualities such as nondestructiveness and easy automation [85]. Becker et al. [86] used ultrasonic methodology in developing a density-measuring device for monitoring beer fermentation. The possibility of obtaining a theoretical relation between density and ultrasonic propagation velocity was rejected by the authors, being aware of the lack of a representative model for liquid mixtures and solutions [87]. Using an ultrasonic velocity measurement technique, Resa et al. [87] studied the alcoholic fermentation of several carbohydrate aqueous solution. From their study, they derived that during glucose (or fructose) fermentation, the density and sound velocity measurements can relate both the sound velocity and the density to the concentration of the main components. Lamberti et al. [85] also presented a similar work for monitoring the alcoholic wine fermentation. They observed a steady decrease in propagation velocity as the fermentation process progressed. They confirmed that the variation of the propagation velocity is exclusively due to the decreasing of the sucrose concentration.

### 4.3. Biosensors

Electrochemical biosensor devices have become more prevalent in the food processing industry in the last 20 years due to their attractive performances. The electrochemical biosensing methods can provide an accurate, highly selective, sensitive, and potentially automatic analysis. It is a widely known fact that the traditional legacy-based analytical techniques are time-consuming and often require specific expensive [88] equipment, hence electrochemical biosensor devices have emerged as a promising alternative. Miniaturization and the development of hand-held devices for real-time monitoring is easily achievable with this technology. Additionally, full automation is being custom developed for continuous online or periodic monitoring of various processes in the food industry [88].

**Figure 6:**
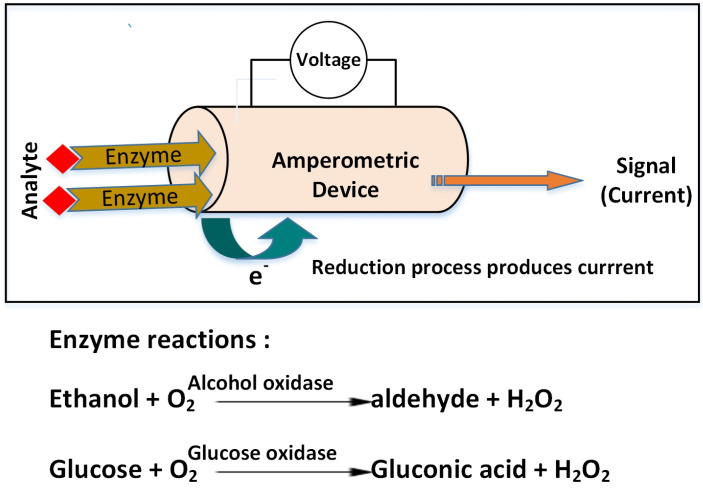
General scheme of amperomertic biosensors for detecting sugar and alcohol levels in process industry.

Most of the time, electrochemical biosensor devices are based on the amperometry principle, where electrochemical oxidation or reduction of an electroactive species produces the current and the resulting current is proportional to the concentration of the analyte. The basic working principle and the key enzyme reactions used in biosensors to determine the analytes are shown in Figure 6. The measurement system generally includes three electrodes (working, reference, and auxiliary electrodes). The working electrode is either metal (Pt and Au) or carbon-based material (glassy carbon, graphite, screen-printed, or carbon paste electrode) and is usually combined with immobilized enzymes [88].

For the online detection of ethanol, glucose, and glycerol in wines, Niculescu et al. [89] proposed dehydrogenase-based biosensors. However, the fabrication process of these devices required isolation and purification of PQQ-dependent dehydrogenase and the synthesis of the electrochemical mediator (PVI13dmeOs), which was a painstaking process. Esti et al. [90] developed conventional amperometric biosensors inserted into flow injection analysis (FIA) systems for detection of glucose, fructose, and ethanol. The system worked effectively for real-time monitoring of alcoholic fermentation in industrial-scale red wine-making. Still, overall, it was somewhat inconvenient to use. To overcome all these limitations, Goriushkina et al. [91] developed amperometric biosensors to detect glucose and ethanol in wine in a more convenient way. The used specific enzymes such as alcohol and glucose oxidase (AO and GOx) in the biosensor. The working potential was chosen to counter high biosensor sensitivity and low current signal due to electrochemical interference [92].

Platinum or carbon (doped with cobalt phthalocyanine) screen-printed electrodes and alcohol oxidase were used to develop other biosensor devices to detect the ethanol in wine and beer [92,93]. These devices are required to accommodate the different strategies to avoid interference that was caused due to the application of high potential. Piermarini et al. [92] used graphite screen-printed sensors modified with Prussian Blue (PB) to develop the biosensors for the detection of both sugars as well as ethanol to overcome the limitation. In addition, an amperometric ferrocene-mediated glucose biosensor with a Nafion protective film operating at 0.25 V (vs. Ag/AgCl, 3 M KCl) was developed to determine the glucose content in wine [94]. Likewise, neutral red [95] and osmium polymers [88] were used as mediators for glucose analysis in wines and alcoholic beverages.

### 4.4. Commercially Available In-Line Brix Sensors

In-line Brix sensing can be effectively used to monitor the progress of the wine-fermentation process. These sensors allow more frequent measurements that can provide data more effectively to winemakers. Based on this data, winemakers can make informed process decisions more frequently and in a much shorter time frame, which improves the productivity and reduces the risk of sluggish ferments. A few such commercially available in-line Brix sensors are discussed in the following paragraphs and summarised in Table 8 and the specifications of discussed in-line Brix sensors are listed in the Table 9.

**Liquiphant M vibrating fork:** This sensor is based on the density measurement principle. It includes a tuning fork that is excited to the resonance frequency. The tuning fork’s oscillating frequency changes according to the density of the liquid in which it is immersed. The shift in frequency is converted into a proportional output signal with the help of a density computing unit. Simultaneously up to four different sensors can be connected to the computing unit [96].

**Fermetrol probe:** A semipermeable water-responsive polymer is used in this sensor to evaluate the osmotic potential caused by the concentration of sugar in the must. As fermentation progresses, alcohol concentration increases. The polymer responds to the increased concentration of alcohol by expansive pressure in the polymer. The expansive pressure of the polymer is converted to an electrical signal with the help of a highly stable ceramic membrane. The output signal can be displayed locally on the unit or fed into a control system [96].

**Micro-LDS:** This sensor determines mass-flow utilizing the Coriolis effect. It consists of a small hollow silicon tube as the sensing element. Vibrating at very high frequencies, the sensor detects variations in fluid density as a shift in tube frequency. The sensor has an integrated temperature sensor to provide the fluid temperature reading. The measured density is converted to Baume and can be displayed locally or fed into a control unit. The sensor also has a solenoid valve and filter mechanism to collect the fermented sample physically [96].

**VS-3000:** This is a solid-state type of sensor based on the absorbance principle. It passes a mid-infrared signal into the fluid and measures the absorbance of the liquid medium. VS-300 has an integrated predefined calibration software to convert the measured signal into a corresponding concentration. The sensor can measure alcohol level, CO_2_, specific gravity, and Baume [96].

**Tilt-hydrometer:** This is a free-floating digital hydrometer specifically designed for home brewing. This instrument allows the reading of the specific gravity and temperature on various Bluetooth devices such as Apple iPhone/iPad or Android smartphone/tablet, or Tilt Pi by downloading the free app on the device. It comes with a calibrated and ready-to-use preinstalled battery. The battery life is approximately 12–14 months, depending on the usage. It is also possible to log the data to the cloud using the Google Sheets template. The accuracy is ±0.002 within the Tilt’s range of 0.990 to 1.120. The thermometer is accurate ±1 ${}^{\circ }$F (±0.5 ${}^{\circ }$C) [101].

## 5. Discussion and Outlook

In this modern era, awareness of health has gained importance in communities across the world. People are more interested in consuming good-quality food products. Hence, grading and sorting of the product have become an essential task in the food industry. Various quality assessment procedures have been implemented with the help of modern technology. In this review paper, different Brix sensors and methods used for sugar measurement in agriculture, vineyards, wineries and breweries have been discussed in detail.

**Figure 7:**
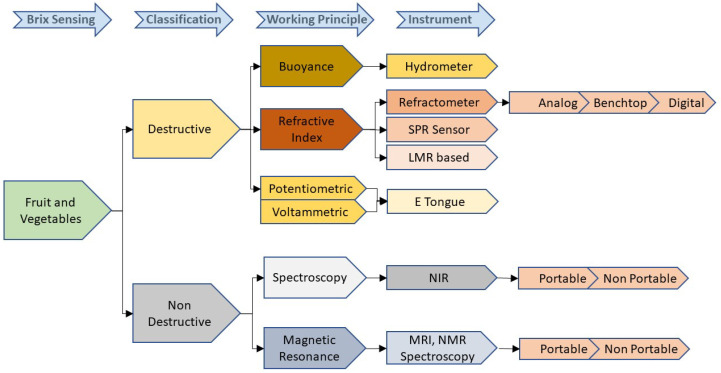
Brix measurement methods for fruits and vegetables.

The methods used to detect the sugar concentration in fruit and vegetables are broadly classified as destructive and non-destructive methods (Figure 7). All the conventional methods such as hydrometry, refractometry, and chromatography come under the umbrella of destructive methods. A few modern technologies such as electronic tongue and SPR-based sensors are also considered to be destructive methods. Though the hydrometer is a widely used, cost-effective, and handheld instrument for Brix measurement, it includes a manual operation that may lead to errors such as the incorrect reading of the meniscus. As its operation is temperature-dependent, temperature compensation has to be incorporated in the final reading. Sometimes, considerable changes in the surface tension due to the surface contamination could lead to additional errors. These limitations are countered by an optical device called a refractometer. This device can be further classified between analog, digital, and benchtop types. An Analog refractometer is a low-cost handheld device that provides a subjective reading and may include some inaccuracies. These drawbacks are covered in digital- and benchtop-type refractometers by compromising on the cost. Electronic tongue is another cost-effective and portable solution for a quick Brix analysis, however, its low selectivity is a potential drawback. Even though most of these destructive techniques are widely used due to their advantages, such as cost-effectiveness, portability, and quick response times, the significant disadvantage of these methods is that the analysis only provides the attributes of the specific product being examined that makes the quality measurement a sample-based evaluation. Another drawback is that the method requires an extract from a fruit or a vegetable for the analysis, resulting in the crushing of the fruits or vegetables. Hence, the use of a nondestructive method becomes a potential requirement in the fruit and vegetable analysis.

Methods such as vis/NIR spectroscopy and magnetic resonance are considered to be widely researched nondestructive techniques. The spectroscopic method is considered to be a candidate method for the nondestructive quality analysis of fruits and vegetables. The recent developments and research in vis/NIR techniques have made it a portable device that can provide rapid and reliable measurements. Hence it is considered to be the best method for nondestructive quality analysis of fruits and vegetables. However, the technique comes with some limitations. It is based on reference values that may be susceptible to errors. Additionally, it requires a new calibration model for every fruit species. The up-gradation of calibration models according to the change in season and location is required as well. On the other hand, though NMR spectroscopy and MRI have been proved effective non-destructive methods for quality evaluation of fruits, they have found only limited use in modern industrial and commercial applications. Currently, the size, weight, cost, and stability of magnets and the throughput are significant constraints for the wider application of MR-based technology.

**Figure 8:**
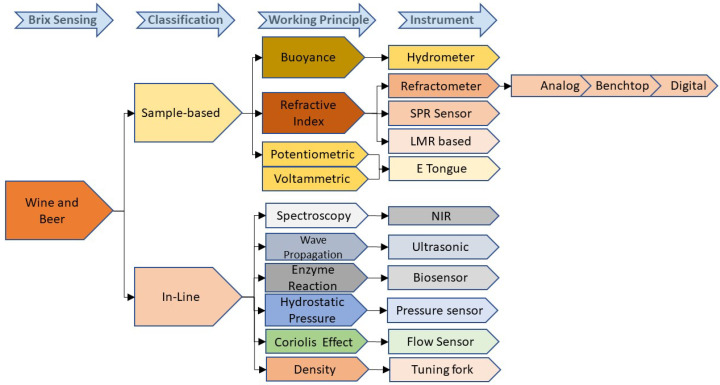
Brix measurement methods for wine and beer.

In wineries and breweries, the fermentation process control plays an important role during the production of wine or beer. Hence, it is necessary to observe the Brix level during beer and wine production stages. Methods for Brix monitoring in the winery and brewery can be categorized as sample-based and in-line monitoring (Figure 8). A hydrometer and a refractometer are traditional sample-based Brix measurement methods used in the winery and brewery. Various research group have developed many electronic methods. The main disadvantage of these methods is that they require a manual sample collection of the liquid from the tank. In a large commercial winery, this tedious job causes a delay in the results and produces chemical waste. Thus, in-line measurement for continuous Brix monitoring is the prime requirement at the winery and brewery.

Currently, in the research community, biosensors are popular to achieve in-line continuous Brix monitoring. The main reason for their popularity is that biosensors do not require extensive sample preparation. A suitable dilution is usually sufficient; the high specificity and wide linear dynamic range allow direct quantification of the target analyte. However, parameters such as the accuracy of measurements, sensor-to-sensor reproducibility, and operational lifetime are substantially influenced by enzyme stability. That means enzyme immobilization is the key factor in the development of efficient biosensors. Sometimes, the adoption of different strategies to eliminate electrochemical interference due to high applied potential is required. Many commercially available in-line measurement methods have been discussed in the paper. Most of them are turned to be an expensive solution for larger wineries. Some of them are complex to install. Keeping the sensor clean during and after fermentation is another problem. Overall, a rapid, sensitive, nondestructive, and in-line Brix monitoring method is currently needed in the industry.

## 6. Conclusions

Brix measurement is an essential part of the quality analysis of agricultural products and alcoholic beverages. Different Brix measurement methods used in the farming industry, winemaking, and brewing, such as destructive, nondestructive, and sample-based, are reviewed here, along with their advantages and disadvantages. Additionally, commercially available in-line measurement Brix measurement techniques are also discussed. Existing methods for measuring the composition and quality of fruit, vegetables, and alcoholic beverages are not adequate for the demands of production in a global market, because of their cost and slow turnaround time. Factors such as promptness and low cost of analysis, minimal sample preparation, and environmentally friendly methods for Brix measurement are being striven towards by the food, winemaking, and brewing industries.

## Figures and Tables

**Table 1:** Summary of refractometers used in various research of horticulture and agriculture products.

Product	Application	Instrument	Model	Ref.
Melon	SSC (%Brix)	Hand Refractometer	Not specified,	[16]
			Atago, Tokyo, Japan	
Blackberry	SSC (%Brix)	Hand Refractometer	Abbe refractometer,	[17]
			Atago, Tokyo, Japan	
Olive	Sugar content (${}^{\circ }$Brix)	Hand Refractometer	PR-32α,	[18]
			Atago, Bellevue, WA, USA	
Apricot	SSC (%Brix)	Digital Refractometer	PR-101,	[19]
			Atago, Nor-folk, VA, USA	
Apricot	SSC (%Brix)	Digital Refractometer	PR-1,	[20]
			Atago, Tokyo, Japan	
Peach	Soluble solids (${}^{\circ }$Brix)	Digital Refractometer	PR-101,	[21]
			Atago, Tokyo, Japan	

SSC: Soluble solid content.

**Table 2:** An overview of commercial refractometers available in the market.

Instrument	Model	Measuring Range	Accuracy	Cost (Approx)	Ref.
Handheld devices					
Analog refractometer	HENAN refractometers	Not specified	Not specified		[22]
Analog refractometer	BRM-100 STARR INSTRUMENTS	0 to 32% Brix	±0.2 Brix%	AUD 99	[23]
Digital refractometer	Brix Meter-BX-1,	0.0 to 85.0% Brix	±0.2 Brix%		[24]
	Bell Technology Ltd.				
Digital refractometer	ATAGO PAL-3880	0 to 33% Brix	Not specified	AUD 395	[25]
Digital refractometer	30GS, Mettler toledo	0 to 100% Brix	±0.2 Brix%	Not specified	[26]
Benchtop devices					
Digital refractometer	Excellence R4, Mettler Toledo	0 to 100% Brix	±0.05 Brix%	USD 5140	[27]
Digital refractometer	Abbemat 450, Anton-Paar	0 to 100% Brix	±0.05 Brix%	Not specified	[28]
Digital refractometer	Laxco™ RBD6000	0 to 100% Brix	±0.05 Brix%	USD 9300	[29]
Digital refractometer	Laxco™ RHDB105	0 to 59.9% Brix	±0.2 Brix%	USD 1526	[30]
Digital refractometer	Anton Paar 173452	0 to 100% Brix	±0.05 Brix%	USD 6960	[31]

**Table 3:** Sample-based destructive Brix sensors reported in the literature.

Sensor	Range	Accuracy	Resolution	Ref.
RI-based optical meter	0–85 (${}^{\circ }$Brix)	Not specified	Not specified	[13]
RI-based SPR sensor	0–25 (${}^{\circ }$Brix)	Not specified	Not specified,	[15]
LMR-based sensor	0–50 (${}^{\circ }$Brix)	Not specified	0.005 ${}^{\circ }$Brix	[32]
Electronic tongue	Not specified	90%	Not specified	[39]

**Table 4:** Summary of NIR methods reported in the literature.

Product	Instrument	Mode	Spectral Range	Detector	No. of Scan	Accuracy	Ref.
Non-portable methods							
Apples	In-house developed	Reflectance	800 to 1600 nm	Not specified	5 to 6	SEP = 0.7322 to 1.7809	[61]
Banana	In-house developed	Reflectance	600 to 870 nm	Photodiode bank	Not specified	97%	[46]
Pomegranate	dual-channel spectrometer,	Reflectance	400 to 1100 nm	Not specified	Not specified	R = 0.94	[44]
	AvaSpec-2048TEC					SEP = 0.24 ${}^{\circ }$Brix	
Apples	Scanning Monochromator	Reflectance	400 to 2500 nm	Silicon	Not specified	R = 0.82	[47]
	Model 6500, NRISystems					SEP = 0.61 ${}^{\circ }$Brix	
Sugarcane	FR, Spectroradiometer	Reflectance	350 to 2500 nm	Not specified	25	R^2^ = 0.76	[48]
Mandarin	CCD spectrometer	Transmittance	350 to 1040 nm	Photodiode array	Not specified	R = 0.92	[49]
	USB4000					SEP = 0.65 ${}^{\circ }$Brix	
Portable Methods							
Pineapple	In house developed	Reflectance	780, 850, 870, 910, and 940 nm	Photodiode	Not specified	80.56%	[50]
Tomato	In house developed	Reflectance	703 to 1124 nm	Photodiode array	Not specified	R = 0.92	[51]
Grapes	In house developed	Reflectance	640 to 1300 nm	Not specified	Not specified	R^2^ = 0.874 to 0.930	[52]
Oranges	In house developed	Reflectance	200 to 1100 nm	CCD	Not specified	R^2^ = 0.918	[53]
						SEP = 0.321 ${}^{\circ }$Brix	
Sugarcane	vis/SW-NIRS,	Reflectance	325 to 1075 nm	Not specified	20	R = 0.87	[54]
	FieldSpec HandHeld					SEP = 1.45 ${}^{\circ }$Brix	
Mango	Fruit Tester 20	Reflectance	600 to 1000 nm	Silicon diode array	30	R = 0.98	[55]
						SEP = 0.40 ${}^{\circ }$Brix	
Nectarines	NIR-Gun, FANTEC	Reflectance	600 to 1100 nm	Silicon diode array	Not specified	R = 0.90	[56]
						SEP = 0.50 ${}^{\circ }$Brix	

SEP: Square errors of prediction.

**Table 5:** Summary of MRI methods reported in various research.

Product	Measured Features	Application	Ref.
Pomegrantate	T2	Measurement of SSC	[67]
Tomato	T1 & T2	Study of tomato ripening stages	[68]
Peach & Apple	T2	Measurement of total sugar content	[69]
Pear	T1 & T2	Study of fruit growing stages	[70]
		Measurement of sugar content	
Grapes	T1 & T2	Study of ripening stages of grape berries	[71]

SSC: Soluble solid content.

**Table 6:** Specifications of nondestructive Brix sensors reported in the literature.

Sensor	Range	Accuracy	Resolution	Ref
NIR-based nonportable sensor	Not specified	97%	Not specified	[46]
NIR-based portable sensor	Not specified	80.56%	Not specified	[50]
MRI-based sensor	Not specified	Not specified	Not specified	[67,68,69]

**Table 7:** Summary of sugar content in different wines using vis-NIR spectroscopy as reported in various research articles.

Parameters	Type of Wine	Wavelength Range nm	Predictions	Ref.
Reducing sugars (g L^−1^)	Red, rose and white wines	400–2500	R^2^ = 0.705	[80]
Sugar (${}^{\circ }$Brix)	White wine	1000–2500	r = 0.99	[81]
Glucose (g L^−1^)	Red wine	400–2500	R^2^ = 0.987	[82]
soluble solids content (${}^{\circ }$Brix)	Rice wine	325–1075	r = 0.95	[83]
Total sugars (g/L)	Red, rose and white wines	1000–2500	R^2^ = 0.94	[84]

**Table 8:** Summary of commercially available in-line Brix sensors.

Sensor	Supplier	Principle	Measurement	Temp Sensor	Approximately Cost	Ref.
Liquiphant M	Endress &	Density	In tank	Extra Required	$2–3K	[96]
vibrating fork	Hauser					
Deltapoint S	Endress &	Hydrostatic	In tank	Not		[97]
	Hauser	pressure		Required	$3K	
Micropilot	Endress &	Volume	Tank header	Not	combined	[96]
	Hauser	(Level)		Required		
Fermetrol	Psitec	Osmotic potential	In tank	Integrated	$2K	[96]
MicroLDS	ISSYS	Mass flow (liquid)	By-pass loop	Integrated	$1K	[96]
VS-3000	Vital Sensors	Absorbance	By-pass loop	Integrated	$3K	[96]
Biosensor	OptiEnz	Enzyme response	By-pass loop	Not Required	$3K	[96]
DT301	Smar technology	Hydrostatic pressure	In tank	Integrated	Not available	[98]
Complete analyzer	Sensotech	Ultrasonic pressure	In tank	Integrated	Not available	[99]
iPR B^3^	SCHMIDT	Absorbance	In tank	Integrated	NZD18K	[100]
	HAENSCH					
Tilt	Tilt Hydrometer	Hydrostatic pressure	Home brewing	Integrated	$250	[101]

**Table 9:** Specifications of in-line Brix sensors.

Sensor	Range	Accuracy	Resolution	Ref.
DT301	0 to 10 g/cm^3^	±0.0004 g/cm^3^	Not specified	[98]
VS-3000	0–20 °Plato	±0.1 ${}^{\circ }$Brix	0.01 ${}^{\circ }$Brix	[102]
MicroLDS	0.6–1.3 g/cc	0.0005 g/cc	0.0001 g/cc	[103]
iPR B^3^	0–90 ${}^{\circ }$Brix	±0.1 ${}^{\circ }$Brix	0.01 ${}^{\circ }$Brix	[104]
Tilt	0.990 to 1.120 of SG	±0.002 of SG	Not specified	[101]

## Abbreviations

The following abbreviations are used in this manuscript:

DM	Dry matter
HPLC	High-performance liquid chromatography
LMR	Lossy mode resonance
PCA	Principal component analysis
RI	Refractive index
SG	Specific gravity
SSC	Soluble Solid Content
TA	Total Acidity
vis/NIRS	Visible/near-infrared spectroscopy
